# Comparing the efficacy of dexamethasone implant and anti-VEGF for the treatment of macular edema: A systematic review and meta-analysis

**DOI:** 10.1371/journal.pone.0305573

**Published:** 2024-07-10

**Authors:** Hui-xin Tang, Jing-jing Li, Ying Yuan, Yun Ling, Zubing Mei, Hong Zou

**Affiliations:** 1 Department of Ophthalmology, Shuguang Hospital Affiliated to Shanghai University of Traditional Chinese Medicine, Shanghai, China; 2 Department of Pediatrics, Shuguang Hospital Affiliated to Shanghai University of Traditional Chinese Medicine, Shanghai, China; 3 Department of Anorectal Surgery, Shuguang Hospital, Shanghai University of Traditional Chinese Medicine, Shanghai, China; 4 Anorectal Disease Institute of Shuguang Hospital, Shanghai, China; Akita University: Akita Daigaku, JAPAN

## Abstract

**Objectives:**

To evaluate the clinical efficacy of dexamethasone (DEX) implant, for the treatment of macular edema (ME) caused by retinal vein occlusion (RVO) and diabetic retinopathy (DR) through a systematic review and meta-analysis.

**Methods:**

The PubMed, Embase and Cochrane Library databases were comprehensively searched from inception to November 21, 2022, for studies evaluating the clinical efficacy of DEX implant for patients with retinal vein occlusion macular edema (RVO-ME) or diabetic macular edema (DME). Randomized controlled trials (RCTs) published in English were considered eligible. The Cochrane Collaboration tool was applied to assess the risk of bias in each study. Effect estimates with 95% confidence intervals (CIs) were pooled using the random effects model. We also conducted subgroup analyses to explore the sources of heterogeneity and the stability of the results.

**Results:**

This meta-analysis included 8 RCTs (RVO-ME [n = 2] and DME [n = 6]) assessing a total of 336 eyes. Compared with anti-VEGF therapy, DEX implant treatment achieved superior outcomes in terms of best corrected visual acuity (BCVA) (mean difference [MD] = -3.68 ([95% CI, -6.11 to -1.25], P = 0.003), and no heterogeneity was observed (P = 0.43, I^2^ = 0%). DEX implant treatment also significantly reduced central macular thickness (CMT) compared with anti-VEGF treatment (MD = -31.32 [95% CI, -57.92 to -4.72], P = 0.02), and there was a high level of heterogeneity between trials (P = 0.04, I^2^ = 54%). In terms of severe adverse events, DEX implant treatment had a higher risk of elevated intraocular pressure than anti-VEGF therapy (RR = 6.98; 95% CI: 2.16 to 22.50; P = 0.001), and there was no significant difference in cataract progression between the two groups (RR = 1.83; 95% CI: 0.63 to 5.27, P = 0.31).

**Conclusions:**

Compared with anti-VEGF therapy, DEX implant treatment is more effective in improving BCVA and reducing ME. Additionally, DEX implant treatment has a higher risk of elevated intraocular pressure. Due to the small number of studies and the short follow-up period, the results should be interpreted with caution. The long-term effects of the two treatments need to be further determined.

**Trial registration:**

**Prospero Registration Number** CRD42021243185.

## Introduction

Macular edema (ME) is an important cause of serious visual impairment. ME can be caused by a variety of eye diseases, of which diabetic retinopathy (DR) and retinal vein occlusion (RVO) are the main causes. Due to changes in living habits, the number of diabetic macular edema (DME) cases caused by DR increases annually. The global caseload of diabetes was estimated to be 463 million in 2019, and it is expected to increase to 578 million by 2030 and to 700 million by 2045 [1]. DR is the most common complication of diabetes, with an incidence rate up to 27%, and is the leading cause of preventable blindness in the working-age population; DME has an incidence rate of 4.6% [2, 3]. RVO is the second most common cause of blindness in retinopathy (second only to DR), with an incidence rate of 0.5%. At present, there are approximately 16 million individuals with RVO worldwide [4]. ME is the most common complication of RVO [5, 6]. Long-term ME can lead to permanent damage to the retinal structure, resulting in persistent vision loss [7]. Therefore, reducing ME is the key to saving the patient’s vision.

Laser and anti-VEGF drugs and steroid drugs are widely used in the treatment of RVO-ME and DME. Laser photocoagulation of microaneurysms and diffuse leaky areas by localized lasers as well as grid lasers can reduce exudation and thus reduce the development of macular edema, but this treatment will inevitably cause permanent damage to the retinal structure, visual field defects, subretinal fibrosis, choroidal neovascularization, and other side effects [8]. At present, the clinical effect of anti-VEGF drugs is remarkable, so they are currently the first choice in the treatment of DME and RVO-ME [9–15]. However, it may cause subconjunctival hemorrhage, endophthalmitis, geographic atrophy, and increase the risk of cardiovascular events in diabetics and the elderly [16].

Current research suggests that ME is driven by a number of factors. The mechanism of RVO-ME is as follows: damage to retinal pigment epithelium (RPE) cell structure and function induced by retinal ischemia and hypoxia and damage to its junction complex [17, 18]. Retinal ischemia induces the expression of monocyte chemoattractant protein-1 (MCP-1) and macrophage inflammatory protein-1α (MCP-1α) to recruit and activate circulating macrophages [19], which in turn activate microglial macrophages to release tumor necrosis factor (TNF-α). TNF-α stimulates the production of interleukin 8 (IL-8), VEGF, basic fibroblast factor (BFGF), MCP-1 and other cytokines [20] by retinal endothelial cells and glial cells, promotes the adhesion of glial cells to microvessels, and induces retinal neovascularization. The overexpression of VEGF and occludin increases the permeability of the vascular endothelium, destroys the inner barrier [11, 21, 22], decreases the expression of occludin tight junction protein [23] and induces the expression of a series of inflammatory factors [22] to destroy the blood retinal barrier. The main mechanism of DME is the change in blood‒retinal barrier (BRB) permeability induced by hyperglycemia [21], such as the increase in endothelial and pericyte apoptosis [24]. Cytokines such as interleukin-6 (IL-6) and tumor necrosis factor-α (TNF-α), immunoglobulin superfamily molecules such as intercellular adhesion molecule-1 (ICAM-1) and vascular cell adhesion molecule-1 (VCAM-1), arachidonic acid and their metabolites, and transcription factors, and inflammatory cells such as leukocytes and neutrophils can destroy the BRB, and fluid and molecules of size can leak through the damaged BRB rupture, causing ME [25]. Because of the multiple factors involved, anti-VEGF drugs are not currently effective in all patients with ME. Studies have shown that approximately 40% of patients with DME [26] and approximately 30% of patients with RVO-ME [27] have no efficacy or response. With the development of molecular mechanisms, increasing attention has been devoted to anti-inflammatory mechanisms. Current studies on ME indicate that overexpression of vascular endothelial growth factor (VEGF) and inflammatory mechanisms play an important role in the development of ME [7]. Both anti-VEGF drugs and dexamethasone (DEX) implants are currently effective in reducing ME and improving best corrected visual acuity (BCVA) [28, 29]. The importance of anti-inflammatory treatment for macular edema secondary to branch retinal vein occlusion (RVO-ME) has also been outlined in the 2019 Guidelines for the Management of Retinal Vein Occlusion by the European Society of Retina Specialists (EURETINA) [30]. Steroids, such as the anti-inflammatory drug DEX, may cause elevated intraocular pressure and cataract progression [31], which restrict the treatment of ME. DEX implants are becoming more widely used in clinical practice, as their development addresses problems such as frequent injections, short duration, and poor compliance. However, due to the small number of samples in clinical trials, its safety and effectiveness have yet to be considered.

To comprehensively evaluate the efficacy and safety of anti-VEGF and DEX implant in the treatment of RVO-ME and DME, we conducted a systematic review since both anti-VEGF drugs (such as ranibizumab and bevacizumab) and DEX implant (such as Ozurdex) can be used to treat RVO-ME and DME in the clinic.

## Methods

The study was conducted in accordance with the Cochrane Handbook for Systematic Reviews and reported using the Preferred Reporting Items for Systematic Review and Meta-Analysis (PRISMA) guidelines. The study also considers PRISMA checklist requirements to the extent possible (S1 Table). The review protocol was registered in the International Prospective Register of Systematic Reviews (PROSPERO) (CRD42021243185).

### Search strategy

The PubMed, Embase and Cochrane Library databases were systematically searched from inception to November 21, 2022, by two independent investigators. We used MeSH (for PubMed and Cochrane)/Emtree (for Embase) terms combined with free-text words (including synonyms and closely related words) that were associated with RVO-ME, DME, DEX and anti-VEGF. The detailed search strategy and specific terms used in the search are shown in **S1 File**. We also manually checked the references of relevant articles, meta-analyses, reviews, and meeting abstracts. We performed study selection in a series of consecutive stages, including duplicate checking using Endnote software, title and abstract screening, and full-text article selection according to the eligibility criteria. These processes were conducted independently by two investigators. Disagreements were resolved by consensus, and a third party was consulted when necessary. If different opinions were encountered, a senior expert was consulted.

### Inclusion and exclusion criteria

The inclusion criteria were as follows: 1. studies were randomized controlled trials (RCTs); 2. studies included patients with RVO-ME or DME; and 3. studies directly compared the clinical efficacy of intravitreal injection of Ozurdex with that of anti-VEGF. We examined the following primary outcomes: 1) best-corrected visual acuity (BCVA), 2) central macular thickness (CMT), 3) cataract, and 4) intraocular pressure (IOP). Additional outcomes collected included the mean number of intravitreal injections. Patients taking bevacizumab and ranibizumab were assigned to the anti-VEGF group. The corresponding authors had to be contacted, and the necessary data were unavailable. The exclusion criteria were as follows: studies with insufficient data; non-RCTs; case reports; review articles; patients in the trial group were given nonsimple Ozurdex injections, including Ozurdex combined with pseudoinjection; or patients in the control group were nonsimple anti-VEGF, such as anti-VEGF combined with pseudoinjection or anti-VEGF combined with laser photocoagulation.

### Data collection and quality assessment

For each trial, the following data were extracted in line with the PICOS framework: first author, year of publication, sample size, interventions, and main outcome indicators. Two authors independently assessed the risk of bias for each RCT according to the recommendation criteria of the Cochrane Handbook for Systematic Reviews of Interventions [32]. There were seven domains that were evaluated, including random sequence generation, allocation concealment, blinding methods (including investigators, participants, and outcome assessment), attrition bias, reporting bias and other sources of bias. Each potential source of bias was evaluated at three levels: high, low or unclear bias. Any disagreements between the two authors were resolved through discussion. Data extraction was also done independently by 2 authors.

### Outcomes of interest

The primary outcomes were as follows: 1) improvement in BCVA (letters) compared with baseline and 2) decrease in CMT (micrometers; μm) compared with baseline. In RCTs, visual acuity (VA) was frequently quantified and reported as an Early Treatment Diabetic Retinopathy Study (ETDRS) letter score. When the logarithm of the minimum angle of resolution (logMAR) or Snellen chart scores were used to measure VA, the score was converted to approximate ETDRS letter scores using the method proposed by Gregori et al. [33], which was used in quantitative analysis.


logMAR=−1xlog(Snellenfraction)

ApproximateETDRSletterscores=85+50xlog(Snellenfraction)

The secondary outcomes were as follows: 1) progression of cataract compared with baseline; and 2) any instance intraocular pressure (IOP) increase at any follow-up visit count compared to baseline IOP.

### Statistical analysis

All statistical analyses were performed using the data statistics software Review Manager (RevMan) version 5.3.

The primary outcome measures were improvement in BCVA from baseline and a decrease in CMT from baseline. The secondary outcomes included the progression of cataracts compared with baseline and IOP compared with baseline.

The random effects model was selected to pool data due to the anticipated high level of heterogeneity, mainly with respect to the varied enrolled populations. The primary outcomes (BCVA and CMT) were expressed as pooled mean differences (MDs) with 95% CIs. The secondary outcomes (IOP and cataracts) were expressed as pooled RRs with 95% CIs.

Between-study heterogeneity was calculated by the I^2^ statistic. I^2^ ≥ 50% indicated substantial heterogeneity [34]. P values < 0.05 were considered statistically significant. To explore the sources of heterogeneity, we carried out a series of subgroup analyses based on the main courses of ME (RVO or DR), different drugs in the control group (bevacizumab or ranibizumab), and follow-up period (≤6 months or >6 months). Sensitivity analysis was performed by applying the leave-one-out method.

## Results

### Literature search and study characteristics

**Fig 1:**
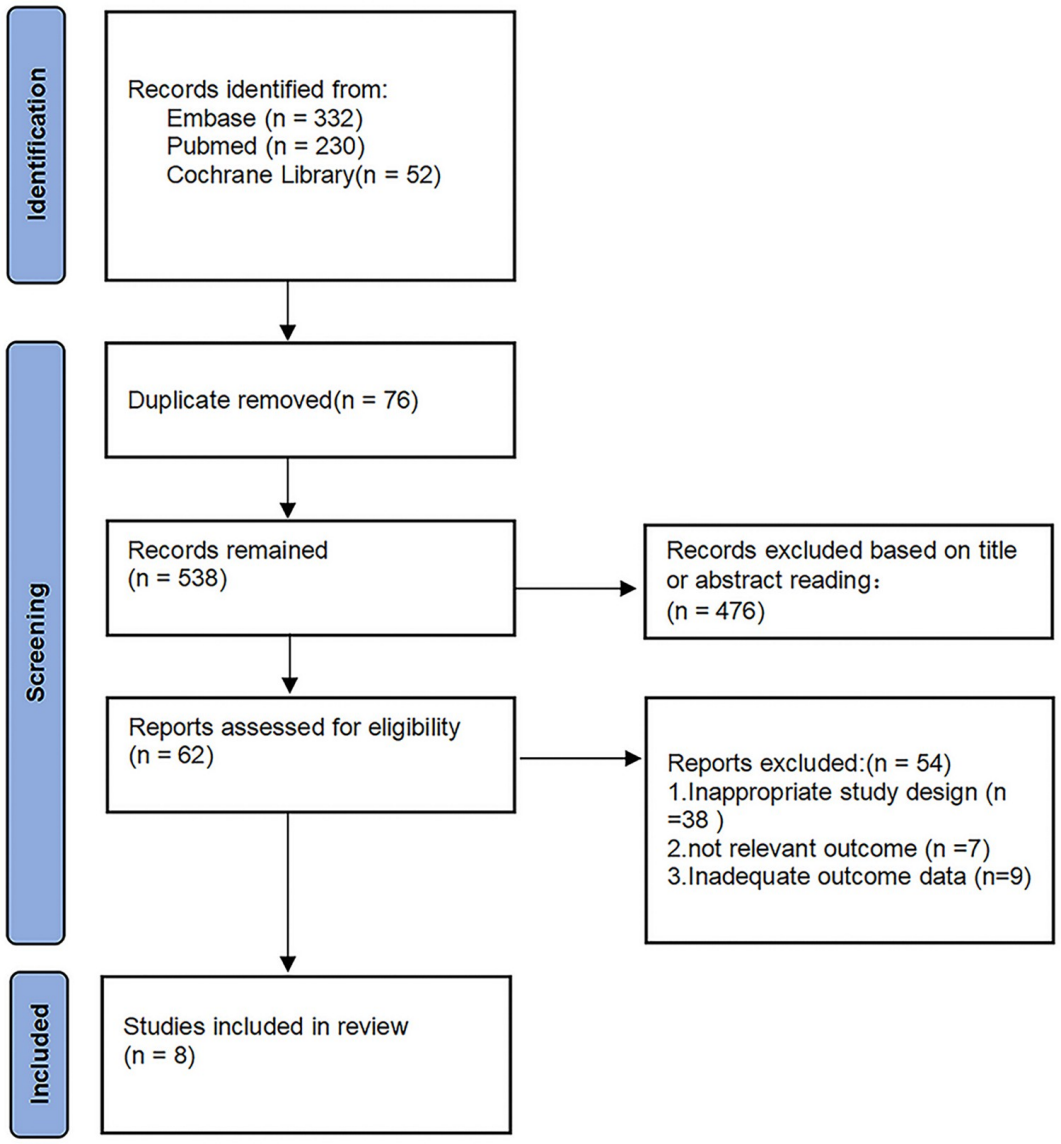
Flow diagram of study selection.

The initial electronic-based literature search identified a total of 614 studies from the Cochrane Library (n = 52), PubMed (n = 230) and Embase (n = 332). After removing duplicates, a total of 538 potentially eligible studies were retrieved. During this process, we excluded 476 studies after reading the titles and abstracts. After reading the full texts, we further excluded 38 studies because of inappropriate study designs. After looking through all eligible studies, 9 studies did not have adequate outcome data, and 7 studies reported irrelevant outcomes. Ultimately, 8 studies [35–42] comprising 336 study eyes satisfied the inclusion criteria and were eligible to be included in the final meta-analysis (**Fig 1**).

**Table 1** presents the baseline characteristics of the included studies. Studies were published between 2012 and 2021 with an average follow-up duration of 8.13 months (range: 4–24 months). In all included studies, 2 studies focused on ME caused by RVO, and 6 studies focused on DME. Five of these studies reported side effects, including increased IOP and cataract progression. All included studies used DEX implant as the treatment group drug and chose anti-VEGF as the control group drug. Among the control groups, 4 used bevacizumab, while 3 used ranibizumab. However, in the study by Sharma et al. that defined only anti-VEGF injection in the control group as bevacizumab or ranibizumab, the authors did not specify the drugs clearly.

**Table 1:** Baseline characteristics of the included trials.

Study (year)	Design	Blinding methods	Subject	Period (months)	Participants numbers		Types and Average number of injections	
					anti-VEGF group	DEX implant group	anti-VEGF group	DEX implant group
**NCT 2012**	RCT	Single blind	DME	6	10 eyes	10 eyes	Bevacizumab	Ozurdex
							5 times	2 times
**Guignier 2013**	RCT	Not mentioned	BRVO-ME	6	8 eyes	11 eyes	Bevacizumab	Ozurdex
							3 times	1 times
**Shah 2016**	RCT	Single blind	DME	7	23 eyes	27 eyes	Bevacizumab	Ozurdex
							7.0±0.5 times	2.7±0.5 times
**Wickremasinghe 2017**	RCT	Single blind	DME	24	22 eyes	22 eyes	Bevacizumab	Ozurdex
							14.2±7.9 times	14.2±7.9 times
**Kumar 2019**	RCT	Not mentioned	BRVO-ME	6	30 eyes	30 eyes	Ranibizumab	Ozurdex
							3 times	1 times
**Podkowinski 2020**	RCT	Double blind	DME	6	9 eyes	9 eyes	Ranibizumab	Ozurdex
							2 times	2 times
**Sharma 2020**	RCT	Not mentioned	DME	6	20 eyes	20 eyes	Bevacizumab or Ranibizumab	Ozurdex
							4.04 times	1.15 times
**Mishra 2021**	RCT	Double blind	DME	4	64 eyes	63 eyes	Ranibizumab	Ozurdex
							3 times	1 times

Abbreviations: RCT = randomized controlled trial; DME = diabetic macular edema; DEX = dexamethasone; RVO-ME = macular edema secondary to branch retinal vein occlusion; VEGF = vascular endothelial growth factor.

### Methodological quality (risk of bias)

The Cochrane Collaboration tool was applied to assess the risk of bias in each study based on the Cochrane Handbook. Random sequence generation and allocation concealment were clear for 8/8 (100%). The RCTs had a low risk of bias with respect to blinding of participants and trial personnel (4/8, 50%), blinding of outcome assessment (3/8, 37.5%), incomplete outcome data (6/8, 75%) and selective reporting (8/8, 100%). Three of the 7 RCTs (42.86%) had an unclear risk of bias, and 1 RCT (12.5%) had a high risk of bias with regard to the other criteria. The risk of bias assessment for each study is shown in **S1 and S2 Figs.**

### Comparison of the effectiveness of DEX implant and Anti-VEGF in DME and RVO-ME

**Fig 2:**
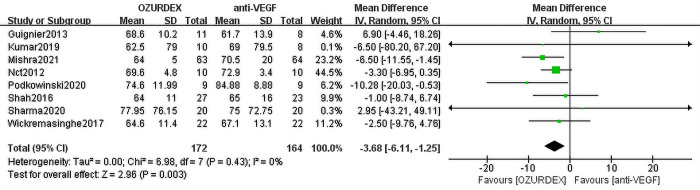
Forest plot for meta-analysis of the effect of ME on BCVA.

**Fig 3:**
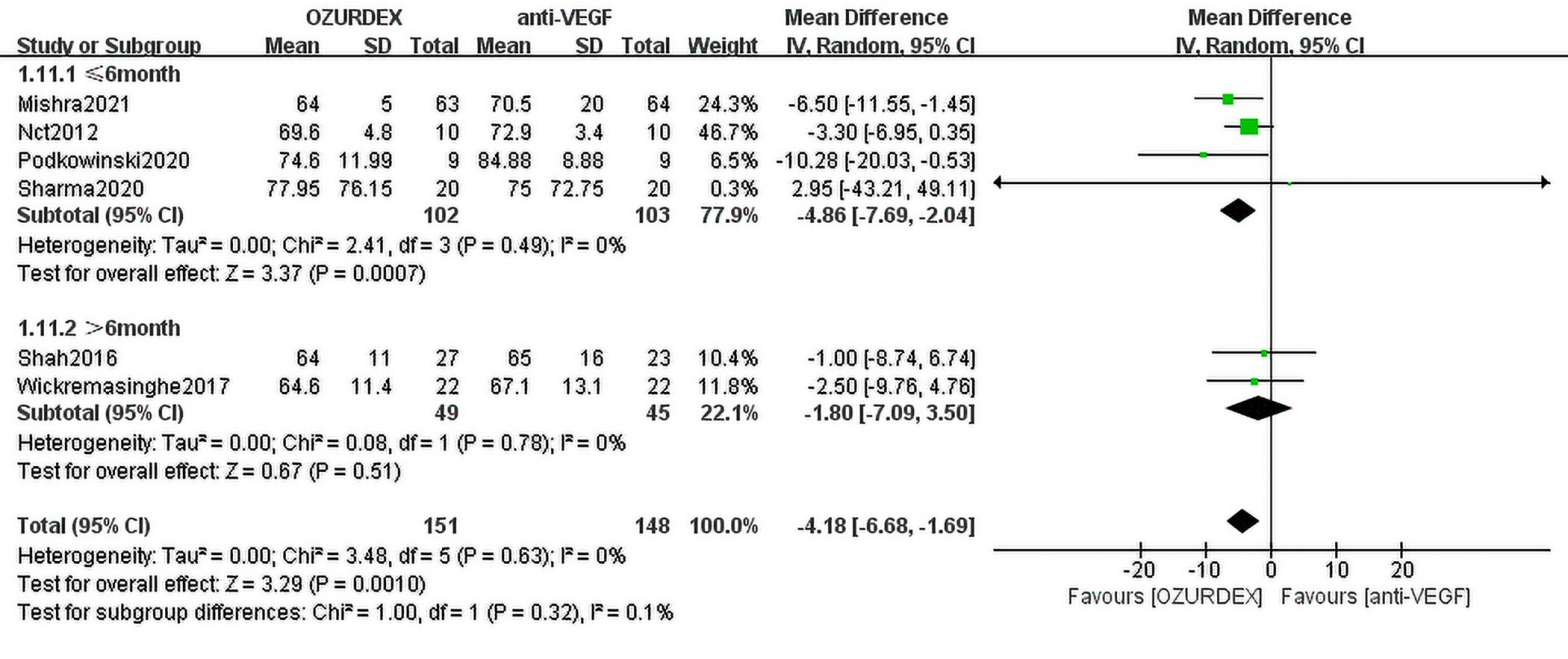
Forest plot for meta-analysis of the effect of ME on CMT.

When we meta-analyzed the 8 studies, the results showed that the pooled MD of BCVA reached -3.68 ([95% CI, -6.11 to -1.25], *P* = 0.003) in the DEX implant treatment group compared with the anti-VEGF treatment groups. No heterogeneity was observed (*P* = 0.43, I^2^ = 0%) (**Fig 2**). Data from the included studies reported that the reduction in central macular thickness (CMT) was significantly greater in the DEX implant group (MD = -31.32 [95% CI, -57.92 to -4.72], *P* = 0.02). Heterogeneity among studies was high (*P* = 0.04, I^2^ = 54%) (**Fig 3**).

## Meta-analysis results

### 1. BCVA

#### 1.1 The effect of treatment modality on BCVA

Eight RCTs assessing 336 eyes (172 eyes with DEX implant treatment, 164 eyes with anti-VEGF treatment) indicated BCVA in patients with RVO-ME or DME. The treatment effect of DEX implant was shown to be better than that of anti-VEGF. The MD in visual acuity of 8 trials was -3.68 ([95% CI, -6.11 to -1.25], *P* = 0.003). No statistical heterogeneity was observed (*P* = 0.43s, I^2^ = 0%) (**Fig 2**).

#### 1.2 Subgroup analysis for the effect of treatment modality on BCVA

Two RCTs of RVO-ME assessing 37 eyes (21 eyes with DEX implant treatment, 16 eyes with anti-VEGF treatment) indicated an improvement in BCVA from baseline. The DEX implant group showed a similar mean change in BCVA from baseline compared with the anti-VEGF group (MD = 6.59; 95% CI, −4.64 to 17.82; *P* = 0.25), and no heterogeneity was observed (*P* = 0.72; I^2^ = 0%) (**Table 2**). A meta-analysis on DME was conducted based on 6 RCTs, including 299 eyes (151 eyes with DEX implant treatment, 148 eyes with anti-VEGF treatment). There were statistically significant differences between the DEX implant and anti-VEGF groups in favor of the DEX implant group (MD = -4.18; [95% CI, -6.68 to -1.69], *P* = 0.001). No statistical heterogeneity was observed (*P* = 0.63, I^2^ = 0%) (**Table 2**).

**Table 2:** Summary results of the subgroup analysis for the effect of treatment modality on BCVA.

Variables	MD	95% CI	I^2^, %	No. of studies
**ME type**				
RVO-ME	6.59	-4.64,17.82	0	2
DME	-4.18	-6.68, -1.69	0	6
**Different drugs**				
Ranibizumab	-7.30	-11.78, -2.82	0	3
Bevacizumab	-2.18	-5.09,0.72	0	4
**Study time**				
≤6 months	-4.20	-7.88, -0.52	20	5
>6 months	-1.80	-7.09,3.50	0	2

Abbreviations: BCVA = best corrected visual acuity; DME = diabetic macular edema; RVO-ME = macular edema secondary to branch retinal vein occlusion; ME = macular edema; CI = confidence interval; MD = mean difference.

Data from 4 RCTs assessing 133 eyes (70 eyes with DEX implant treatment, 63 eyes with bevacizumab treatment) indicated an improvement in BCVA from baseline. There were no statistically significant differences between the DEX implant and bevacizumab groups (MD = -2.18 [95% CI, -5.09 to 0.72], *P* = 0.14), and no significant heterogeneity was observed (*P* = 0.41, I^2^ = 0%) (**Table 2**). Data from 3 studies assessing 163 eyes (82 eyes with DEX implant treatment, 81 eyes with ranibizumab treatment) indicated an improvement in BCVA from baseline. Compared with ranibizumab, DEX implant treatment was superior (MD = -7.30 [95% CI, -11.78 to -2.82], *P* = 0.001). No statistical heterogeneity was observed (*P* = 0.80, I^2^ = 0%) (**Table 2**).

Data from 5 RCTs with a follow-up period ≤ 6 months assessing 242 eyes (123 eyes with DEX implant treatment, 119 eyes with anti-VEGF treatment) indicated an improvement in BCVA from baseline. There were statistically significant differences between the DEX implant and anti-VEGF groups (MD = -4.20 [95% CI, -7.88 to -0.52], *P* = 0.03). A low level of heterogeneity was observed (*P* = 0.28, I^2^ = 20%) (**Table 2**). Data from 2 studies with a follow-up period > 6 months assessing 94 eyes (49 eyes with DEX implant treatment, 45 eyes with anti-VEGF treatment) indicated an improvement in BCVA from baseline. There were no statistically significant differences between the DEX implant and anti-VEGF groups (MD = -1.80 [95% CI, -7.09 to 3.50], *P* = 0.51). No statistical heterogeneity was observed (*P* = 0.78, I^2^ = 0%) (**Table 2**).

#### 1.3 Subgroup analysis of different follow-up periods with BCVA in DME

**Fig 4:**
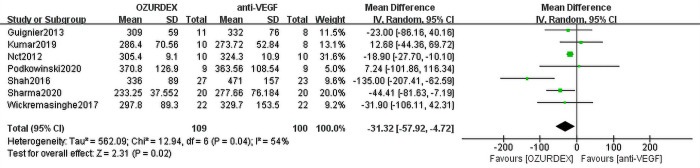
Subgroup analysis of different follow-up periods with BCVA in DME.

Data from 4 RCTs with a follow-up period ≤ 6 months assessing 205 eyes (102 eyes with DEX implant treatment, 103 eyes with anti-VEGF treatment) indicated an improvement in BCVA from baseline in DME. There were statistically significant differences between the DEX implant and anti-VEGF groups, and the MD in visual acuity of the 4 trials was -4.86 ([95% CI, -7.69 to -2.04], *P* = 0.007) (**Fig 4**). No statistical heterogeneity was observed (*P* = 0.49, I^2^ = 0%). Two RCTs with a follow-up period >6 months assessing 94 eyes (49 eyes with DEX implant treatment, 45 eyes with anti-VEGF treatment) included data on the change in BCVA. No difference was found in the two studies (MD = -1.80 [95% CI, -7.09 to 3.50], *P* = 5.01), and no statistical heterogeneity was observed (*P* = 0.78, I^2^ = 0%) (**Fig 4**).

### 2. CMT

#### 2.1 The effect of treatment modality on CMT

Data from 7 RCTs assessing 209 eyes (109 eyes with DEX implant treatment, 100 eyes with anti-VEGF treatment) indicated a reduction in CMT from baseline. Meta-analysis demonstrated that the DEX implant group showed a remarkable reduction in CMT from baseline in the DEX implant group. The MD for all studies was statistically significant (MD = -31.32 [95% CI, -57.92 to -4.72], *P* = 0.02) in favor of DEX implant treatment over anti-VEGF treatment, and there was a high level of heterogeneity (*P* = 0.04, I^2^ = 54%) (**Fig 3**).

#### 2.2 Subgroup analysis for the effect of treatment modality on CMT

Data from 2 RCTs of RVO-ME assessing 37 eyes (21 eyes with DEX implant treatment, 16 eyes with anti-VEGF treatment) indicated a reduction in CMT from baseline. The DEX implant group reported a similar mean change in CMT from baseline compared with the anti-VEGF group (MD = -3.35 ([95% CI, -45.68 to 38.98], *P* = 0.88), and no heterogeneity was observed (*P* = 0.41, I^2^ = 0%) (**Table 3**). A meta-analysis on DME was conducted based on 5 RCTs, including 172 eyes (88 eyes with DEX implant treatment, 84 eyes with anti-VEGF treatment). There were statistically significant differences between the DEX implant and anti-VEGF groups in favor of the DEX implant group (MD = -42.36 [95% CI, -77.99 to -6.72], *P* = 0.02). A high level of heterogeneity was observed (*P* = 0.02, I^2^ = 65%) (**Table 3**).

**Table 3:** Summary results of the subgroup analysis for the effect of treatment modality on CMT.

Variables	MD	95% CI	I^2^, %	No. of studies
**ME type**				
RVO-ME	-3.35	-45.68,38.98	0	2
DME	-42.36	-77.99, -6.72	65	5
**Different drugs**				
Ranibizumab	11.51	-39.03,62.06	0	2
Bevacizumab	-45.54	-92.18,1.10	69	4
**Study time**				
≤6 months	-19.43	-27.80, -11.06	0	5
>6 months	-83.78	-184.82,17.25	74	2

Abbreviations: CMT = central macular thickness; DME = diabetic macular edema; RVO-ME = macular edema secondary to branch retinal vein occlusion; ME = macular edema; CI = confidence interval; MD = mean difference.

Data from 4 RCTs assessing 133 eyes (70 eyes with DEX implant treatment, 63 eyes with bevacizumab treatment) indicated a reduction in CMT from baseline. There were no statistically significant differences between the DEX implant and bevacizumab groups (MD = -45.54 [95% CI, -92.18 to 1.10], *P* = 0.06)), and a high level of heterogeneity was observed (*P* = 0.02, I^2^ = 69%) (**Table 3**). Data from 2 studies assessing 36 eyes (19 eyes with DEX implant treatment, 17 eyes with ranibizumab treatment) indicated a reduction in CMT from baseline. No statistically significant differences between the DEX implant and ranibizumab groups were found (MD = 11.51 [95% CI, -39.03 to 62.06], *P* = 0.66), and no statistical heterogeneity was observed (*P* = 0.93, I^2^ = 0%) (**Table 3**).

Data from 5 RCTs with a follow-up period ≤6 months assessing 115 eyes (60 eyes with DEX implant treatment, 55 eyes with anti-VEGF treatment) indicated a reduction in CMT from baseline. The MD for the 5 studies was statistically significant (MD = -19.43 [95% CI, -27.80 to -11.06], *P*<0.00001) in favor of DEX implant treatment over anti-VEGF treatment, and no heterogeneity was observed (*P* = 0.50, I^2^ = 0%) (**Table 3**). Data from 2 studies with a follow-up period > 6 months assessing 94 eyes (49 eyes with DEX implant treatment, 45 eyes with anti-VEGF treatment) indicated a reduction in CMT from baseline. There was no statistically significant difference between the DEX implant and anti-VEGF groups (MD = -83.78 [95% CI, -184.82 to 17.25], *P* = 0.10). A high level of heterogeneity was observed (*P* = 0.05, I^2^ = 74%) (**Table 3**).

#### 2.3 Subgroup analysis of different follow-up periods with CMT in DME

**Fig 5:**
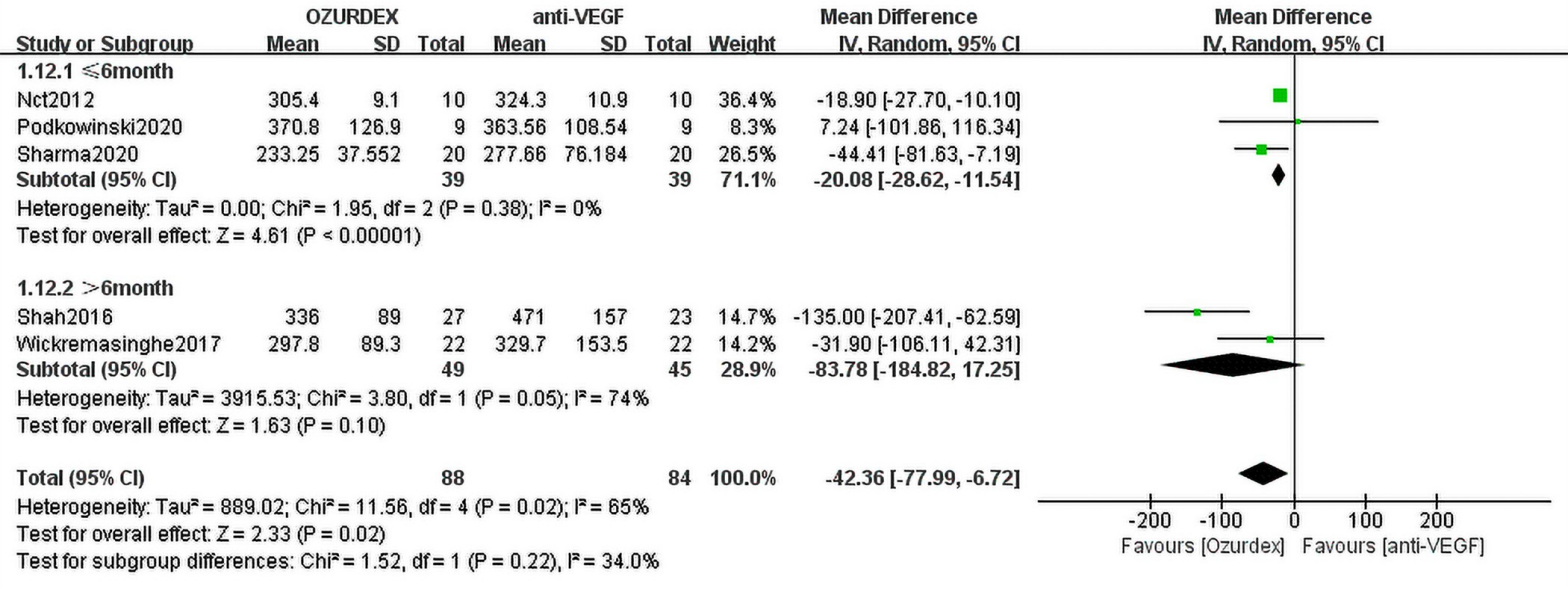
Subgroup analysis of different follow-up periods with CMT in DME.

Data from 3 RCTs with a follow-up period ≤6 months assessing 78 eyes (39 eyes with DEX implant treatment, 39 eyes with anti-VEGF treatment) indicated a reduction in CMT from baseline in DME, the treatment effect of DEX implant is better than that of anti-VEGF. The MD in CMT of 3 trials was -20.08 ([95% CI, -28.62 to -11.54], *P*<0.00001) (**Fig 5**), No statistical heterogeneity was observed (*P* = 0.38, I^2^ = 0%). Data from two RCTs with a follow-up period > 6 months assessed CMT in 94 eyes (49 eyes with DEX implant treatment, 45 eyes with anti-VEGF treatment), No difference was found in the two studies (MD = -83.78 [95% CI, -184.82 to 17.25], *P* = 0.10), A high level of heterogeneity was observed (*P* = 0.05, I^2^ = 74%) (**Fig 5**).

### Adverse events

#### IOP

**Fig 6:**
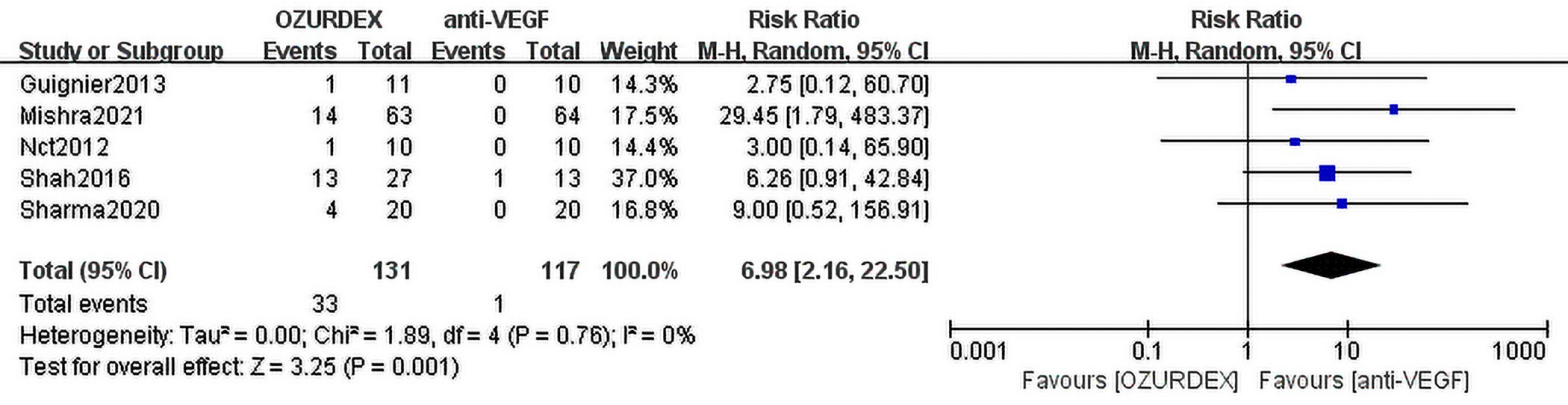
Forest plot for meta-analysis of the effect of elevated IOP.

Five RCTs demonstrated increased IOP after injection of DEX implant/anti-VEGF, NCT et al [35] reported people with elevated IOP, Guignier et al [36] reported people with elevated IOP above normal IOP, Shah et al [37] reported on people with elevated IOP but no more than 5mmHg, Sharma et al [41] reported people with elevated IOP to more than 25, Mishra et al [42] reported people with elevated IOP greater than 15mmHg.NCT et al [35] only reported elevated IOP, did not describe treatment after elevated IOP, and the remaining four studies showed that elevated IOP was controlled with medication. A low level of heterogeneity was observed between studies (I^2^ = 0%, *P* = 0.76). A random effects model demonstrated a statistically significant difference between DEX implant and anti-VEGF treatment (RR = 6.98; 95% CI: 2.16 to 22.50; *P* = 0.001) (**Fig 6**).

#### Progression of cataract

**Fig 7:**
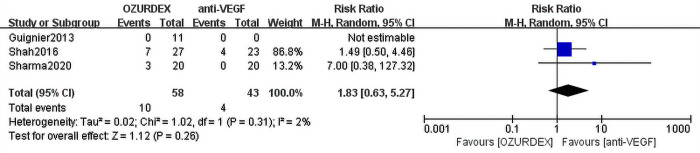
Forest plot for meta-analysis of the effect of cataract progression.

Three studies involving 101 eyes reported postoperative cataracts. No significant difference was found between the DEX implant and anti-VEGF groups (RR = 1.83; 95% CI: 0.63 to 5.27, *P* = 0.31), and no heterogeneity was observed (*P* = 0.26, I^2^ = 2%) (**Fig 7**).

#### Sensitivity analyses and publication bias

Sensitivity analyses were performed using the leave-one-out method to further examine the stability of the results. We found that no individual study significantly altered the summary MDs of BCVA (lowest MD = -2.83, 95% CI, -5.60 to -0.05; highest MD = -4.19, 95% CI, -6.681 to -1.70) and CMT (lowest MD = -19.98, 95% CI, -27.90 to -11.12; highest MD = -38.43, 95% CI, -68.13 to -8.74). Due to the small number of trials included in each meta-analysis, we did not conduct a publication bias test.

## Discussion

### Principal findings

The results of this meta-analysis, which pooled data from 8 RCTs, suggest that in the treatment of ME caused by RVO and DR, DEX implant is superior to anti-VEGF (bevacizumab, ranibizumab) for enhancing BCVA and reducing CMT. The results remained consistent after conducting sensitivity analysis using the leave-one-out method. Subgroup analysis revealed that DEX implant was superior to ranibizumab for improving BCVA, and there was no difference between DEX implant and ranibizumab in reducing CMT. Moreover, the anti-inflammatory treatment effect of DEX implant was superior to that of bevacizumab and ranibizumab in DME, and the RVO-ME results of the two treatments were not different. DEX implant was more effective in reducing ME than the anti-VEGF treatment when the follow-up period was ≤6 months than when the follow-up period was >6 months, regardless of whether ME was caused by DR, RVO or DR alone. The incidence of IOP in the DEX implant group was higher than that in the anti-VEGF drug group, and there was no significant difference between the two treatments in terms of cataract progression.

### Comparisons with previous studies

Our findings are different from the results of a meta-analysis of 4 RCTs and 12 real-world studies that was published last year, which suggested that DEX implants, compared with anti-VEGF agents, had inferior functional efficacy and safety but required fewer injections in RVO patients [43]. In contrast, we found that as a DEX implant, DEX implant treatment is more effective than anti-VEGF therapy in improving BCVA and reducing ME. On the other hand, DEX implant treatment leads to a higher risk of elevated intraocular pressure. He et al [44] studied DME and included the Allergan trial; they only reported differences before and after treatment. Therefore, this trial was excluded from the current meta-analysis. We included the latest clinical research trials such as those reported by Wickremasinghe et al [38], Podkowinski et al [40], Sharma et al [41], and Mishra et al [42]. Retrospective studies were included in Chi et al.’s [45] study and only included patients with DME. This meta-analysis included RCTs in patients with DME and RVO. Pranata et al. [46] meta-analysis included retrospective studies in patients with RVO, ranibizumab in the control group. This meta-analysis included RCTs in patients with DME and RVO with bevacizumab and ranibizumab in the control groups. Although Qiu et al. [47] included both patients with DME and RVO in our study, retrospective studies were included in the meta-analysis by Qiu et al. [47]. This meta-analysis included 8 RCTs (RVO-ME [n = 2] and DME [n = 6]) assessing a total of 336 eyes from PubMed, Embase and the Cochrane Library. However, DR and RVO are both retinal vascular diseases and were analyzed together in this meta-analysis to investigate the efficacy of DEX implant and anti-VEGF in the treatment of ME caused by retinal vascular disease. Furthermore, we compared the difference between anti-VEGF drugs and conducted subgroup analyses of the effect of ME on BCVA and CMT to perform a comprehensive systematic review and meta-analysis.

### Potential mechanisms

Current studies have shown that after RVO, increased capillary pressure leads to increased vascular leakage, local blood formation turbulence damages the vascular endothelium, thrombosis forms, and inflammation [48]. After the inflammatory reaction, RPE cells are degranulated by mast cells and express Toll-like receptors. After Toll-like receptors are expressed, Lipase induces Nuclear Factor Kappa-B activation, which activates the downstream inflammatory pathway [49]. Retinal ischemia induced by RVO induces the expression of MCP-1 and MIP-1α and the recruitment and activation of circulating macrophages [11]. Activated macrophages release TNF-α, and TNF-α stimulates the synthesis of IL-8, VEGF, BFGF, MCP-1 and other cytokines in retinal endothelial cells and glial cells [22] and activates the downstream inflammatory pathway. Inflammatory mechanisms have also been shown to be activated in DME by multiple pathways. For example, retinal hypoxia-activating cytokines such as IL-6 and TNF-α [27] in diabetic patients with long-term hyperglycemia and tumor necrosis factor can induce the expression of ICAM-1 [50] and increase the expression of ICAM-1 and VCAM-1, which can induce leukocyte stasis, microthrombosis and endothelial cell apoptosis. On the other hand, the aggregation of leukocytes on the surface of the retinal capillary can lead to the upregulation of ICAM-1, which mediates the attraction of the endothelium to monocytes and neutrophils [51, 52].

DEX implant can block the production of its inflammatory mechanism through a variety of mechanisms, including reducing the synthesis of inflammatory mediators, reducing the synthesis of VEGF [53], downregulating selectin and integrin to prevent leukocyte arrest [54], and preventing the occurrence of an inflammatory cascade reaction from multiple links. DEX implant directly affects the expression of tight junction proteins and enhances the barrier integrity of retinal endothelial cells by upregulating the expression of Claudin-5 and occludin [55], thus reducing the increase in paracellular permeability caused by protein phosphorylation of endothelial molecules, IL-6, etc. DEX implant inhibited the expression of ICAM-1 in the retina [56], blocked the attraction of ICAM-1-mediated endothelium to monocytes and neutrophil cells, and reduced leukocyte arrest and endothelial cell injury. DEX implant can inhibit the leakage of blood vessels, prevent the osmotic expansion of Müller cells [57], protect their water transport function and reduce edema.

We found that although some of the inflammatory pathways of RVO-ME and DME were blocked by DEX implant, in our meta-analysis, the DEX implant group achieved better efficacy than the anti-VEGF group in treating DME. Combined with previous studies by Arroba, Valverde, Roy and Yu [58–60], oxidative stress and inflammation in the retina induced by chronic hyperglycemia constitute an early stage in the development of DME. We hypothesized that the inflammatory reaction mechanism plays a more important role in DME than VEGF and that the anti-inflammatory mechanism plays a more important role in the treatment of DME than anti-VEGF drugs. However, more robust clinical evidence should be provided before confirming this hypothesis. Therefore, future studies on DME inflammation mechanisms are of great importance in the prevention and treatment of DME.

### Strengths and limitations

The current study has several strengths. First, we included RCTs in the meta-analysis instead of studies with other designs, as they were regarded as high-quality evidence for the estimation of study effects. Second, we developed systematic and comprehensive database search strategies for major online databases (PubMed, Embase and Cochrane Library) with no search date restriction to avoid the impact of publication bias on the pooled findings and improve the repeatability of the results. Third, the evidence quality of all included outcomes was evaluated based on the Cochrane Collaboration’s tool. Fourth, several approaches, including subgroup analyses and sensitivity analyses, were applied to thoroughly determine sources of heterogeneity based on the abstracted study-level baseline characteristics.

Our study is limited by the following factors. First, there were only 8 RCTs (336 eyes) included, and the number of RVO-ME studies was relatively small (n = 2), which was less than the number of DME studies (n = 6). Second, the overall follow-up of the included studies was short. Because through the analysis, we found that the difference between Ozudex and anti-VEGF appeared to be mainly driven by studies ≤ 6 months of follow-up, and no differences were found in studies > 6 months of follow-up. Third, in some clinical trials, the follow-up period of the study was short, which may understate the adverse events caused by the drug, and some of the studies did not document the group of adverse events in detail, which affected our assessment of the incidence of adverse events. Fourth, because the anti-VEGF group had different kinds of drugs and different injection frequencies, DEX implant also had different application frequencies, and the heterogeneity of the study was inevitable. Therefore, more experimental studies are needed to confirm the efficacy and safety of these two treatment strategies.

## Conclusions

In summary, this systematic review and meta-analysis shows that despite some ocular adverse events, DEX implant-treated eyes have relatively superior anatomic outcomes compared with anti-VEGF-treated eyes: DEX implant treatment improves BCVA and reduces CMT significantly in DME. Although anti-VEGF drugs are still the first choice for the treatment of ME, DEX implants may be a good first choice for ME patients who are not sensitive to anti-VEGF drug response, patients with pseudolens eyes, and patients with a low risk of IOP.

## Supporting information

S1 Table PRISMA 2020 checklist. (DOCX)

S1 File Search strategy for each database. (DOCX)

S1 Fig Risk of bias summary. (TIF)

S2 Fig Risk of bias graph. (TIF)

## List of abbreviations

BFGF: basic fibroblast factor
BCVA: best corrected visual acuity
BRB: blood‒retinal barrier
CMT: central macular thickness
CIs: confidence intervals
DEX: dexamethasone
DME: diabetic macular edema
DR: diabetic retinopathy
ETDRS: Early Treatment Diabetic Retinopathy Study
ICAM-1: intercellular adhesion molecule-1
IL-8: interleukin 8
IOP: intraocular pressure
MCP-1α: macrophage inflammatory protein-1α
ME: macular edema
MDs: mean differences
MCP-1: monocyte chemoattractant protein-1
PRISMA: Preferred Reporting Items for Systematic Review and Meta-Analysis
RCTs: randomized controlled trials
RPE: retinal pigment epithelium
RR: risk ratio
RVO: retinal vein occlusion
RVO-ME: macular edema secondary to branch retinal vein occlusion
TNF-α: tumor necrosis factor
VCAM-1: vascular cell adhesion molecule-1
VA: visual acuity
VEGF: vascular endothelial growth factor
