# Supplementary material for: Comparing the efficacy of dexamethasone implant and anti-VEGF for the treatment of macular edema: A systematic review and meta-analysis
Source: PLoS One. 2024 Jul 10;19(7):e0305573. doi: 10.1371/journal.pone.0305573 (PMC11236136; doi:10.1371/journal.pone.0305573)
Supplement: S1 File — (DOCX) [file pone.0305573.s002.docx]

Text S1 Search strategy

**Database: embase from inception to Present> (Search date: November 21, 2022)**

**Search Strategy:**

--------------------------------------------------------------------------------

***DME/RVO-ME terms:***

1 'diabetes mellitus'/exp

2 'diabetic retinopathy'/exp

3 'diabetic retinopathies':ab,ti

4 'retinopathies, diabetic':ab,ti

5 'retinopathy, diabetic':ab,ti

6 'retina vein occlusion'/exp

7 'occlusion, retinal vein':ab,ti

8 'retinal vein occlusions':ab,ti

9 'vein occlusion, retinal':ab,ti

10 'retinal vein thrombosis':ab,ti

11 'retinal vein thromboses':ab,ti

12 'vein thrombosis, retinal':ab,ti

13 'thrombosis, retinal vein':ab,ti

14 'central retinal vein occlusion':ab,ti

15 'branch vein occlusion':ab,ti

16 'branch vein occlusions':ab,ti

17 'occlusion, branch vein':ab,ti

18 'vein occlusion, branch':ab,ti

19 'retinal branch vein occlusion':ab,ti

20 'diabetic complication'/exp

21 'diabetes complications':ab,ti

22 'diabetes complication':ab,ti

23 'diabetes-related complications':ab,ti

24 'diabetes related complications':ab,ti

25 'diabetes-related complication':ab,ti

26 'diabetic complications':ab,ti

27 'complications of diabetes mellitus':ab,ti

28 'diabetes mellitus complication':ab,ti

29 'diabetes mellitus complications':ab,ti

30 OR/1-29

31 'macular edema'/exp

32 'retina maculopathy'/exp

33   31 OR 32

34   30 AND 33

***Dexamethasone terms:***

35 'dexamethasone'/exp

36 'methylfluorprednisolone':ab,ti

37 'hexadecadrol':ab,ti

38 'decameth':ab,ti

39 'decaspray':ab,ti

40 'dexasone':ab,ti

41 'dexpak':ab,ti

42 'maxidex':ab,ti

43 'millicorten':ab,ti

44 'oradexon':ab,ti

45 'decaject':ab,ti

46 'decaject-l.a':ab,ti

47 'decaject l.a.':ab,ti

48 'hexadrol':ab,ti

49 'ozurdex':ab,ti

50 OR/35-49

***Anti-VEGF terms:***

51 'angiogenesis inhibitor'/exp

52 'angiogenesis inhibitors':ab,ti

53 'inhibitor, angiogenesis':ab,ti

54 'angiogenetic antagonist':ab,ti

55 'antagonist, angiogenetic':ab,ti

56 'angiogenetic antagonists':ab,ti

57 'antagonists, angiogenetic':ab,ti

58 'angiogenetic inhibitor':ab,ti

59 'inhibitor, angiogenetic':ab,ti

60 'angiogenetic inhibitors':ab,ti

61 'angiogenic antagonists':ab,ti

62 'angiogenic antagonist':ab,ti

63 'antagonist, angiogenic':ab,ti

64 'angiogenic inhibitor':ab,ti

65 'inhibitor, angiogenic':ab,ti

66 'angiostatic agent':ab,ti

67 'agent, angiostatic':ab,ti

68 'anti-angiogenetic agent':ab,ti

69 'agent, anti-angiogenetic':ab,ti

70 'anti angiogenetic agent':ab,ti

71 'angiogenic inhibitors':ab,ti

72 'angiostatic agents':ab,ti

73 'agents, angiostatic':ab,ti

74 'antagonists, angiogenic':ab,ti

75 'anti-angiogenetic agents':ab,ti

76 'agents, anti-angiogenetic':ab,ti

77 'anti angiogenetic agents':ab,ti

78 'anti-angiogenic drugs':ab,ti

79 'anti angiogenic drugs':ab,ti

80 'drugs, anti-angiogenic':ab,ti

81 'antiangiogenic agents':ab,ti

82 'agents, antiangiogenic':ab,ti

83 'inhibitors, angiogenesis':ab,ti

84 'inhibitors, angiogenetic':ab,ti

85 'inhibitors, angiogenic':ab,ti

86 'inhibitors, neovascularization':ab,ti

87 'neovascularization inhibitors':ab,ti

88 'anti-angiogenic drug':ab,ti

89 'anti angiogenic drug':ab,ti

90 'drug, anti-angiogenic':ab,ti

91 'neovascularization inhibitor':ab,ti

92 'inhibitor, neovascularization':ab,ti

93 'antiangiogenic agent':ab,ti

94 'agent, antiangiogenic':ab,ti

95 'angiogenesis factor inhibitors':ab,ti

96 'factor inhibitors, angiogenesis':ab,ti

97 'inhibitors, angiogenesis factor':ab,ti

98 'angiogenesis factor inhibitor':ab,ti

99 'factor inhibitor, angiogenesis':ab,ti

100 'inhibitor, angiogenesis factor':ab,ti

101 'anti-angiogenesis effect':ab,ti

102 'anti angiogenesis effect':ab,ti

103 'effect, anti-angiogenesis':ab,ti

104 'antiangiogenesis effect':ab,ti

105 'effect, antiangiogenesis':ab,ti

106 'antiangiogenesis effects':ab,ti

107 'effects, antiangiogenesis':ab,ti

108 'anti-angiogenesis effects':ab,ti

109 'anti angiogenesis effects':ab,ti

110 'effects, anti-angiogenesis':ab,ti

111 OR/51-110

112 'angiogenic factor'/exp

113 'angiogenesis inducing agents':ab,ti

114 'agents, angiogenesis inducing':ab,ti

115 'inducing agents, angiogenesis':ab,ti

116 'angiogenesis stimulating agents':ab,ti

117 'agents, angiogenesis stimulating':ab,ti

118 'stimulating agents, angiogenesis':ab,ti

119 'angiogenesis stimulators':ab,ti

120 'stimulators, angiogenesis':ab,ti

121 'angiogenesis inducers':ab,ti

122 'inducers, angiogenesis':ab,ti

123 'angiogenesis factor':ab,ti

124 'factor, angiogenesis':ab,ti

125 'factor, angiogenic':ab,ti

126 'tumor angiogenic factor':ab,ti

127 'angiogenic factor, tumor':ab,ti

128 'factor, tumor angiogenic':ab,ti

129 'angiogenesis effect':ab,ti

130 'effect, angiogenesis':ab,ti

131 'angiogenesis effects':ab,ti

132 'effects, angiogenesis':ab,ti

133 OR/112-132

134 'endothelial cell growth factor'/exp

135 'growth factors, endothelial':ab,ti

136 'endo-gf':ab,ti

137 'endothelial growth factor polypeptides':ab,ti

138 'endothelial growth factor':ab,ti

139 'growth factor, endothelial':ab,ti

140 'ecdgf':ab,ti

141 'endothelial cell-derived growth factors':ab,ti

142 'endothelial cell derived growth factors':ab,ti

143 'beta-endothelial growth factor':ab,ti

144 'growth factor, beta-endothelial':ab,ti

145 'beta endothelial growth factor':ab,ti

146 'alpha-endothelial growth factor':ab,ti

147 'growth factor, alpha-endothelial':ab,ti

148 'alpha endothelial growth factor':ab,ti

149 OR/134-148

150 'macugen':ab,ti

151 'pegaptanib':ab,ti

152 'lucentis':ab,ti

153 'rhufab':ab,ti

154 'ranibizumab':ab,ti

155 'bevacizumab':ab,ti

156 'avastin':ab,ti

157 'aflibercept':ab,ti

158 OR/150-157

159 'vegf trap':ab,ti

160 111 OR 133 OR 149 OR 158 OR 159

***Study design terms:***

1. 'clinical':ti,ab AND 'trial':ti,ab OR 'clinical trial'/exp OR random* OR 'drug therapy':lnk

***Final search results: Combining DME/rvo-me and Dexamethasone and anti-VEGF and Study design:***

162 34 AND 50 AND 160 AND 161 （332）

Text S2 Search strategy

**Database: cochrane from inception to Present> (Search date: November 21, 2022)**

**Search Strategy:**

--------------------------------------------------------------------------------

***DME/RVO-ME terms:***

#1 MeSH descriptor: [Diabetes Mellitus] explode all trees

#2 MeSH descriptor: [Diabetic Retinopathy] explode all trees

#3 (Diabetic Retinopathies):ti,ab,kw OR (Retinopathies, Diabetic):ti,ab,kw OR (Retinopathy, Diabetic):ti,ab,kw (Word variations have been searched)

#4 MeSH descriptor: [Diabetes Complications] explode all trees

#5 (Diabetes Complication):ti,ab,kw OR (Diabetes-Related Complications):ti,ab,kw OR (Diabetes Related Complications):ti,ab,kw OR (Diabetes-Related Complication):ti,ab,kw OR (Diabetic Complications):ti,ab,kw (Word variations have been searched)

#6 (Diabetic Complication):ti,ab,kw OR (Complications of Diabetes Mellitus):ti,ab,kw OR (Diabetes Mellitus Complication):ti,ab,kw OR (Diabetes Mellitus Complications):ti,ab,kw (Word variations have been searched)

#7 MeSH descriptor: [Retinal Vein Occlusion] explode all trees

#8 (Vein Thrombosis, Retinal):ti,ab,kw OR (Thrombosis, Retinal Vein):ti,ab,kw OR (Central Retinal Vein Occlusion):ti,ab,kw OR (Branch Vein Occlusion):ti,ab,kw OR (Branch Vein Occlusions):ti,ab,kw (Word variations have been searched)

#9 (Occlusion, Retinal Vein):ti,ab,kw OR (Retinal Vein Occlusions):ti,ab,kw OR (Vein Occlusion, Retinal):ti,ab,kw OR (Retinal Vein Thrombosis):ti,ab,kw OR (Retinal Vein Thromboses):ti,ab,kw (Word variations have been searched)

#10 (Branch Vein Occlusions):ti,ab,kw OR (Occlusion, Branch Vein):ti,ab,kw OR (Vein Occlusion, Branch):ti,ab,kw OR (Retinal Branch Vein Occlusion):ti,ab,kw (Word variations have been searched)

#11 #1 or #2 or #3 or #4 or #5 or #6 or #7 or #8 or #9 or #10

#12 MeSH descriptor: [Macular Edema] explode all trees

#13 (retina maculopathy):ti,ab,kw (Word variations have been searched)

#14 #12 or #13

#15 #11 and #14

***Dexamethasone terms:***

#16 MeSH descriptor: [Dexamethasone] explode all trees

#17 (methylfluorprednisolone):ti,ab,kw OR (hexadecadrol):ti,ab,kw OR (decameth):ti,ab,kw OR (decaspray):ti,ab,kw OR (dexasone):ti,ab,kw (Word variations have been searched)

#18 (dexpak):ti,ab,kw OR (maxidex):ti,ab,kw OR (millicorten):ti,ab,kw OR (oradexon):ti,ab,kw OR (decaject):ti,ab,kw (Word variations have been searched)

#19 (decaject-l.a):ti,ab,kw OR (decaject l.a.):ti,ab,kw OR (hexadrol):ti,ab,kw OR (ozurdex):ti,ab,kw (Word variations have been searched)

#20 #16 or #17 or #18 or #19

***Anti-VEGF terms:***

#21 MeSH descriptor: [Angiogenesis Inhibitors] explode all trees

#22 (angiogenesis inhibitors):ti,ab,kw OR (inhibitor, angiogenesis):ti,ab,kw OR (angiogenetic antagonist):ti,ab,kw OR (antagonist, angiogenetic):ti,ab,kw OR (angiogenetic antagonists):ti,ab,kw (Word variations have been searched)

#23 (antagonists, angiogenetic):ti,ab,kw OR (angiogenetic inhibitor):ti,ab,kw OR (inhibitor, angiogenetic):ti,ab,kw OR (angiogenetic inhibitors):ti,ab,kw OR (angiogenic antagonists):ti,ab,kw (Word variations have been searched)

#24 (angiogenic antagonist):ti,ab,kw OR (antagonist, angiogenic):ti,ab,kw OR (angiogenic inhibitor):ti,ab,kw OR (inhibitor, angiogenic):ti,ab,kw OR (angiostatic agent):ti,ab,kw (Word variations have been searched)

#25 (agent, angiostatic):ti,ab,kw OR (anti-angiogenetic agent):ti,ab,kw OR (agent, anti-angiogenetic):ti,ab,kw OR (anti angiogenetic agent):ti,ab,kw OR (angiogenic inhibitors):ti,ab,kw (Word variations have been searched)

#26 (angiostatic agents):ti,ab,kw OR (agents, angiostatic):ti,ab,kw OR (antagonists, angiogenic):ti,ab,kw OR (anti-angiogenetic agents):ti,ab,kw OR (agents, anti-angiogenetic):ti,ab,kw (Word variations have been searched)

#27 (anti angiogenetic agents):ti,ab,kw OR (anti-angiogenic drugs):ti,ab,kw OR (anti angiogenic drugs):ti,ab,kw OR (drugs, anti-angiogenic):ti,ab,kw OR (antiangiogenic agents):ti,ab,kw (Word variations have been searched)

#28 (agents, antiangiogenic):ti,ab,kw OR (inhibitors, angiogenesis):ti,ab,kw OR (inhibitors, angiogenetic):ti,ab,kw OR (inhibitors, angiogenic):ti,ab,kw OR (inhibitors, neovascularization):ti,ab,kw (Word variations have been searched)

#29 (neovascularization inhibitors):ti,ab,kw OR (anti-angiogenic drug):ti,ab,kw OR (anti angiogenic drug):ti,ab,kw OR (rug, anti-angiogenic):ti,ab,kw OR (neovascularization inhibitor):ti,ab,kw (Word variations have been searched)

#30 (inhibitor, neovascularization):ti,ab,kw OR (antiangiogenic agent):ti,ab,kw OR (agent, antiangiogenic):ti,ab,kw OR (angiogenesis factor inhibitors):ti,ab,kw OR (factor inhibitors, angiogenesis):ti,ab,kw (Word variations have been searched)

#31 (inhibitors, angiogenesis factor):ti,ab,kw OR (angiogenesis factor inhibitor):ti,ab,kw OR (inhibitor, angiogenesis factor):ti,ab,kw OR (anti-angiogenesis effect):ti,ab,kw OR (factor inhibitor, angiogenesis):ti,ab,kw (Word variations have been searched)

#32 (anti angiogenesis effect):ti,ab,kw OR (effect, anti-angiogenesis):ti,ab,kw OR (antiangiogenesis effect):ti,ab,kw OR (effect, antiangiogenesis):ti,ab,kw OR (antiangiogenesis effects):ti,ab,kw (Word variations have been searched)

#33 (effects, antiangiogenesis):ti,ab,kw OR (anti-angiogenesis effects):ti,ab,kw OR (anti angiogenesis effects):ti,ab,kw OR (effects, anti-angiogenesis):ti,ab,kw (Word variations have been searched)

#34 #21 or #22 or #23 or #24 or #25 or #26 or #27 or #28 or #29 or #30 or #31 or #32 or #33

#35 MeSH descriptor: [Angiogenesis Inducing Agents] explode all trees

#36 (angiogenesis inducing agents):ti,ab,kw OR (agents, angiogenesis inducing):ti,ab,kw OR (inducing agents, angiogenesis):ti,ab,kw OR (angiogenesis stimulating agents):ti,ab,kw OR (agents, angiogenesis stimulating):ti,ab,kw (Word variations have been searched)

#37 (stimulating agents, angiogenesis):ti,ab,kw OR (angiogenesis stimulators):ti,ab,kw OR (stimulators, angiogenesis):ti,ab,kw OR (angiogenesis inducers):ti,ab,kw OR (inducers, angiogenesis):ti,ab,kw (Word variations have been searched)

#38 (angiogenesis factor):ti,ab,kw OR (factor, angiogenesis):ti,ab,kw OR (factor, angiogenic):ti,ab,kw OR (tumor angiogenic factor):ti,ab,kw OR (angiogenic factor, tumor):ti,ab,kw (Word variations have been searched)

#39 (factor, tumor angiogenic):ti,ab,kw OR (angiogenesis effect):ti,ab,kw OR (effect, angiogenesis):ti,ab,kw OR (angiogenesis effects):ti,ab,kw OR (effects, angiogenesis):ti,ab,kw (Word variations have been searched)

#40 #35 or #36 or #37 or #38 or #39

#41 MeSH descriptor: [Endothelial Growth Factors] explode all trees

#42 (endothelial cell growth factor):ti,ab,kw OR (growth factors, endothelial):ti,ab,kw OR (endo-gf):ti,ab,kw OR (endothelial growth factor polypeptides):ti,ab,kw OR (endothelial growth factor):ti,ab,kw (Word variations have been searched)

#43 (growth factor, endothelial):ti,ab,kw OR (ecdgf):ti,ab,kw OR (endothelial cell-derived growth factors):ti,ab,kw OR (endothelial cell derived growth factors):ti,ab,kw OR (beta-endothelial growth factor):ti,ab,kw (Word variations have been searched)

#44 (growth factor, beta-endothelial):ti,ab,kw OR (beta endothelial growth factor):ti,ab,kw OR (alpha-endothelial growth factor):ti,ab,kw OR (growth factor, alpha-endothelial):ti,ab,kw OR (alpha endothelial growth factor):ti,ab,kw (Word variations have been searched)

#45 #41 or #42 or #43 or #44

#46 (macugen):ti,ab,kw OR (pegaptanib):ti,ab,kw OR (lucentis):ti,ab,kw OR (rhufab):ti,ab,kw OR (ranibizumab):ti,ab,kw (Word variations have been searched)

#47 (bevacizumab):ti,ab,kw OR (avastin):ti,ab,kw OR (aflibercept):ti,ab,kw OR (vegf trap):ti,ab,kw (Word variations have been searched)

#48 #46 or #47

#49 #34 or #40 or #45 or #48

***Final search results: Combining DME/rvo-me and Dexamethasone and anti-VEGF and Study design:***

#50 #15 and #20 and #49（52）

Text S3 Search strategy

**Database: pubmed from inception to Present> (Search date: November 21, 2022)**

**Search Strategy:**

--------------------------------------------------------------------------------

***DME/RVO-ME terms:***

1"Diabetes Mellitus"[MeSH Terms] OR "Diabetic Retinopathy"[MeSH Terms] OR "Retinal Vein Occlusion"[MeSH Terms]

2 (((((((((((((((diabetic retinopathies[Title/Abstract]) OR (retinopathies, diabetic[Title/Abstract])) OR (retinopathy, diabetic[Title/Abstract])) OR (occlusion, retinal vein[Title/Abstract])) OR (retinal vein occlusions[Title/Abstract])) OR (vein occlusion, retinal[Title/Abstract])) OR (retinal vein thrombosis[Title/Abstract])) OR (retinal vein thromboses[Title/Abstract])) OR (vein thrombosis, retinal[Title/Abstract])) OR (thrombosis, retinal vein[Title/Abstract])) OR (central retinal vein occlusion[Title/Abstract])) OR (branch vein occlusion[Title/Abstract])) OR (branch vein occlusions[Title/Abstract])) OR (occlusion, branch vein[Title/Abstract])) OR (vein occlusion, branch[Title/Abstract])) OR (retinal branch vein occlusion[Title/Abstract])

3"Diabetes Complications"[MeSH Terms]

4((((((((diabetes complications[Title/Abstract]) OR (diabetes complication[Title/Abstract])) OR (diabetes-related complications[Title/Abstract])) OR (diabetes related complications[Title/Abstract])) OR (diabetes-related complication[Title/Abstract])) OR (diabetic complications[Title/Abstract])) OR (complications of diabetes mellitus[Title/Abstract])) OR (diabetes mellitus complication[Title/Abstract])) OR (diabetes mellitus complications[Title/Abstract])

5 or/1-4

6 "Macular Edema"[MeSH Terms]

7retina maculopathy[Title/Abstract]

8 or/6-7

9 5 and 8

***Dexamethasone terms:***

10 "Dexamethasone"[Mesh]

11 (((((((((((((methylfluorprednisolone[Title/Abstract]) OR (hexadecadrol[Title/Abstract])) OR (decameth[Title/Abstract])) OR (decaspray[Title/Abstract])) OR (dexasone[Title/Abstract])) OR (dexpak[Title/Abstract])) OR (maxidex[Title/Abstract])) OR (millicorten[Title/Abstract])) OR (oradexon[Title/Abstract])) OR (decaject[Title/Abstract])) OR (decaject-l.a[Title/Abstract])) OR (decaject l.a.[Title/Abstract])) OR (hexadrol[Title/Abstract])) OR (ozurdex[Title/Abstract])

12 or/10-11

***Anti-VEGF terms:***

13 "Angiogenesis Inhibitors"[Mesh]

14 ((((((((((((((((((((((((((((((((((((((((angiogenesis inhibitors[Title/Abstract]) OR (inhibitor, angiogenesis[Title/Abstract])) OR (angiogenetic antagonist[Title/Abstract])) OR (antagonist, angiogenetic[Title/Abstract])) OR (angiogenetic antagonists[Title/Abstract])) OR (antagonists, angiogenetic[Title/Abstract])) OR (angiogenetic inhibitor[Title/Abstract])) OR (inhibitor, angiogenetic[Title/Abstract])) OR (angiogenetic inhibitors[Title/Abstract])) OR (angiogenic antagonists[Title/Abstract])) OR (angiogenic antagonist[Title/Abstract])) OR (antagonist, angiogenic[Title/Abstract])) OR (angiogenic inhibitor[Title/Abstract])) OR (inhibitor, angiogenic[Title/Abstract])) OR (angiostatic agent[Title/Abstract])) OR (agent, angiostatic[Title/Abstract])) OR (anti-angiogenetic agent[Title/Abstract])) OR (agent, anti-angiogenetic[Title/Abstract])) OR (anti angiogenetic agent[Title/Abstract])) OR (angiogenic inhibitors[Title/Abstract])) OR (angiostatic agents[Title/Abstract])) OR (agents, angiostatic[Title/Abstract])) OR (antagonists, angiogenic[Title/Abstract])) OR (anti-angiogenetic agents[Title/Abstract])) OR (agents, anti-angiogenetic[Title/Abstract])) OR (anti angiogenetic agents[Title/Abstract])) OR (anti-angiogenic drugs[Title/Abstract])) OR (anti angiogenic drugs[Title/Abstract])) OR (drugs, anti-angiogenic[Title/Abstract])) OR (antiangiogenic agents[Title/Abstract])) OR (agents, antiangiogenic[Title/Abstract])) OR (inhibitors, angiogenesis[Title/Abstract])) OR (inhibitors, angiogenetic[Title/Abstract])) OR (inhibitors, angiogenic[Title/Abstract])) OR (inhibitors, neovascularization[Title/Abstract])) OR (neovascularization inhibitors[Title/Abstract])) OR (anti-angiogenic drug[Title/Abstract])) OR (anti angiogenic drug[Title/Abstract])) OR (drug, anti-angiogenic[Title/Abstract])) OR (neovascularization inhibitor[Title/Abstract])) OR (inhibitor, neovascularization[Title/Abstract])

15 (((((((((((((((((antiangiogenic agent[Title/Abstract]) OR (agent, antiangiogenic[Title/Abstract])) OR (angiogenesis factor inhibitors[Title/Abstract])) OR (factor inhibitors, angiogenesis[Title/Abstract])) OR (inhibitors, angiogenesis factor[Title/Abstract])) OR (angiogenesis factor inhibitor[Title/Abstract])) OR (factor inhibitor, angiogenesis[Title/Abstract])) OR (inhibitor, angiogenesis factor[Title/Abstract])) OR (anti-angiogenesis effect[Title/Abstract])) OR (anti angiogenesis effect[Title/Abstract])) OR (effect, anti-angiogenesis[Title/Abstract])) OR (antiangiogenesis effect[Title/Abstract])) OR (effect, antiangiogenesis[Title/Abstract])) OR (antiangiogenesis effects[Title/Abstract])) OR (effects, antiangiogenesis[Title/Abstract])) OR (anti-angiogenesis effects[Title/Abstract])) OR (anti angiogenesis effects[Title/Abstract])) OR (effects, anti-angiogenesis[Title/Abstract])

16 "Angiogenesis Inducing Agents"[Mesh]

17 (((((((((((((((((((angiogenic factor[Title/Abstract]) OR (agents, angiogenesis inducing[Title/Abstract])) OR (inducing agents, angiogenesis[Title/Abstract])) OR (angiogenesis stimulating agents[Title/Abstract])) OR (agents, angiogenesis stimulating[Title/Abstract])) OR (stimulating agents, angiogenesis[Title/Abstract])) OR (angiogenesis stimulators[Title/Abstract])) OR (stimulators, angiogenesis[Title/Abstract])) OR (angiogenesis inducers[Title/Abstract])) OR (inducers, angiogenesis[Title/Abstract])) OR (angiogenesis factor[Title/Abstract])) OR (factor, angiogenesis[Title/Abstract])) OR (factor, angiogenic[Title/Abstract])) OR (tumor angiogenic factor[Title/Abstract])) OR (angiogenic factor, tumor[Title/Abstract])) OR (factor, tumor angiogenic[Title/Abstract])) OR (angiogenesis effect[Title/Abstract])) OR (effect, angiogenesis[Title/Abstract])) OR (angiogenesis effects[Title/Abstract])) OR (effects, angiogenesis[Title/Abstract])

18 "Endothelial Growth Factors"[Mesh]

19 ((((((((((((((endothelial cell growth factor[Title/Abstract]) OR (growth factors, endothelial[Title/Abstract])) OR (endo-gf[Title/Abstract])) OR (endothelial growth factor polypeptides[Title/Abstract])) OR (endothelial growth factor[Title/Abstract])) OR (growth factor, endothelial[Title/Abstract])) OR (ecdgf[Title/Abstract])) OR (endothelial cell-derived growth factors[Title/Abstract])) OR (endothelial cell derived growth factors[Title/Abstract])) OR (beta-endothelial growth factor[Title/Abstract])) OR (growth factor, beta-endothelial[Title/Abstract])) OR (beta endothelial growth factor[Title/Abstract])) OR (alpha-endothelial growth factor[Title/Abstract])) OR (growth factor, alpha-endothelial[Title/Abstract])) OR (alpha endothelial growth factor[Title/Abstract])

20((((((((macugen[Title/Abstract]) OR (pegaptanib[Title/Abstract])) OR (lucentis[Title/Abstract])) OR (rhufab[Title/Abstract])) OR (ranibizumab[Title/Abstract])) OR (bevacizumab[Title/Abstract])) OR (avastin[Title/Abstract])) OR (aflibercept[Title/Abstract])) OR (vegf trap[Title/Abstract])

21 or/13-20

***Study design terms:***

22 (clinical[tiab] AND trial[tiab]) OR "clinical trials as topic"[mesh] OR "clinical trial"[pt] OR random*[tiab] OR "random allocation"[mesh] OR "therapeutic use"[sh]

***Final search results: Combining DME/rvo-me and Dexamethasone and anti-VEGF and Study design:***

23 9 and 12 and 21 and 22 (230)
