# Supplementary figures and images for: Comparing the efficacy of dexamethasone implant and anti-VEGF for the treatment of macular edema: A systematic review and meta-analysis
Source: PLoS One. 2024 Jul 10;19(7):e0305573. doi: 10.1371/journal.pone.0305573 (PMC11236136; doi:10.1371/journal.pone.0305573)

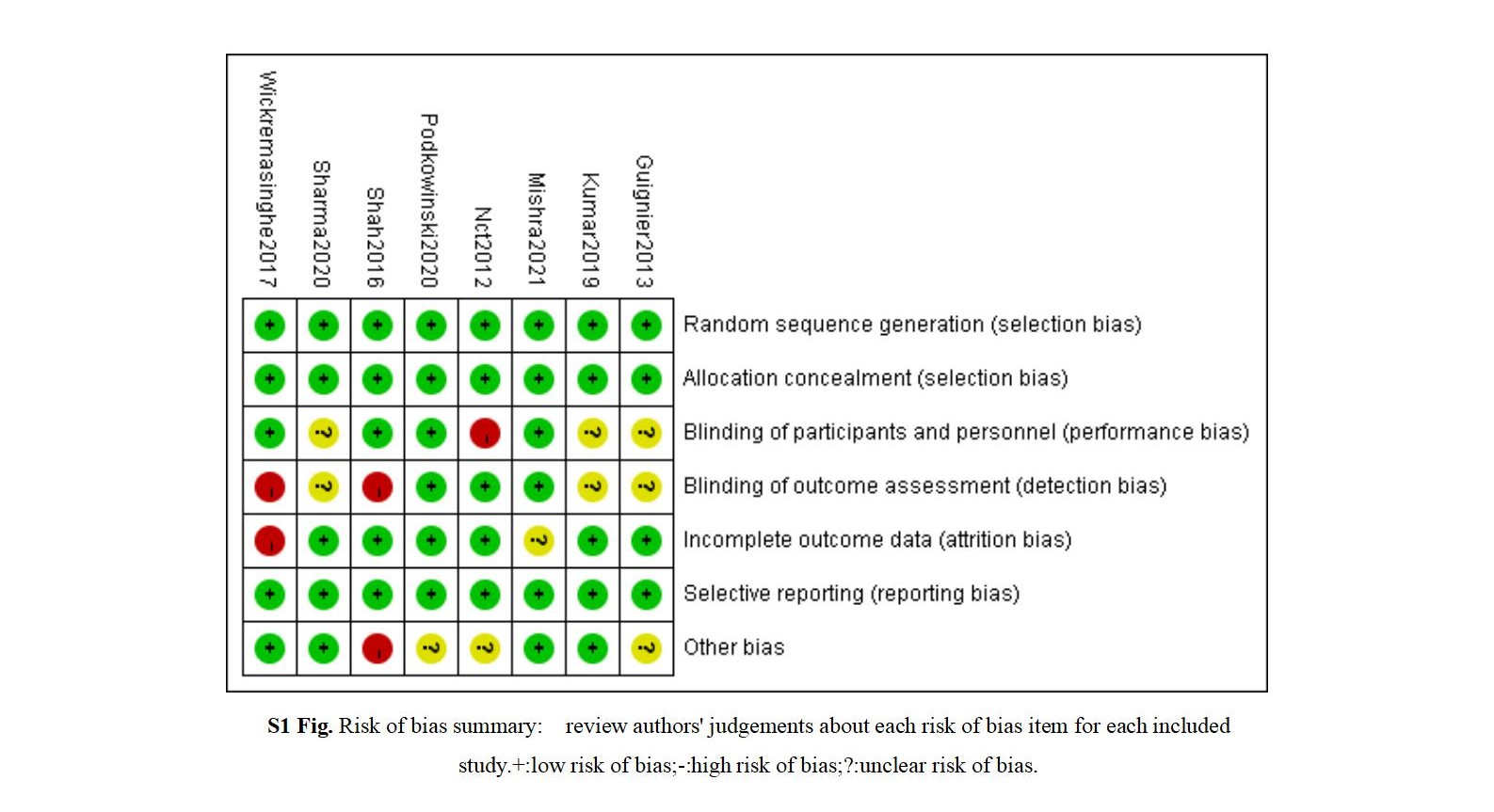

Supplement: S1 Fig — (TIF) [file pone.0305573.s003.tif]

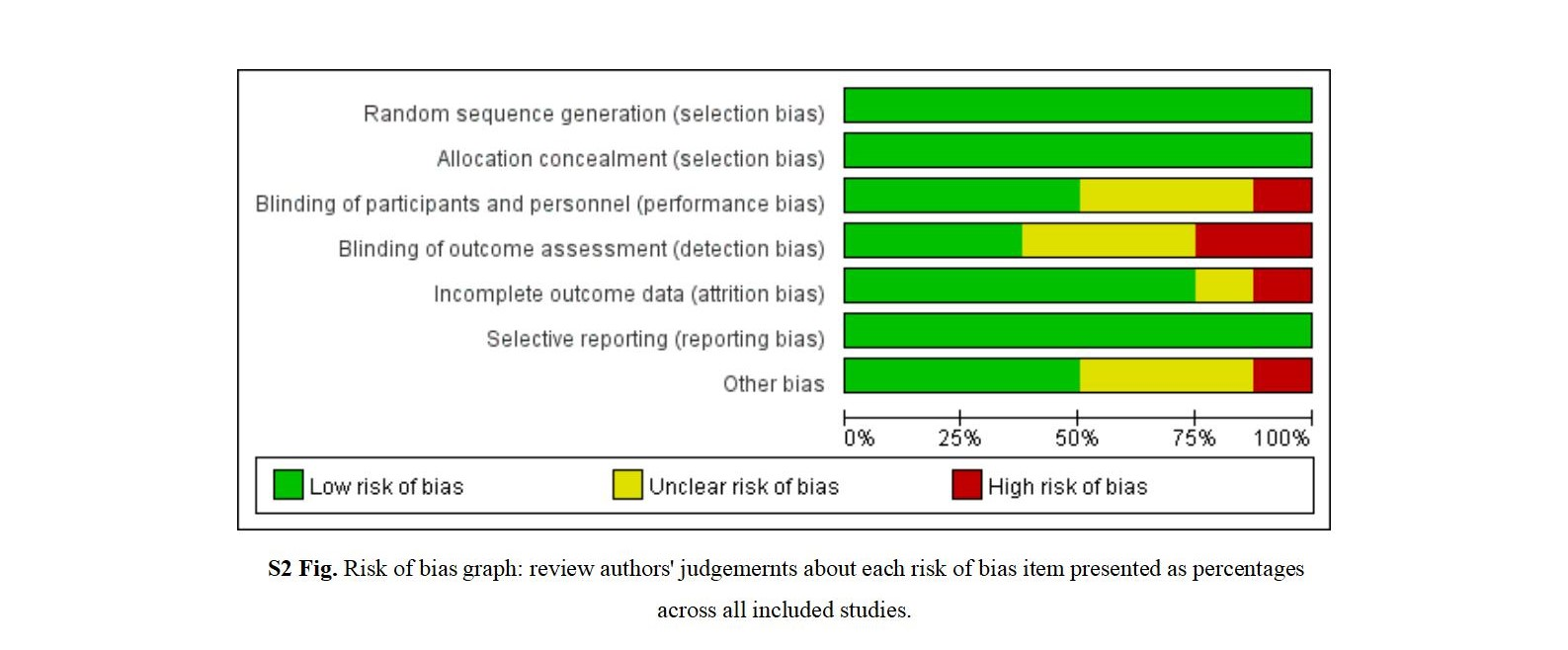

Supplement: S2 Fig — (TIF) [file pone.0305573.s004.tif]
